# LncRNA loc339803 acts as CeRNA of miR-30a-5p to promote the migration and invasion of hepatocellular carcinoma cells

**DOI:** 10.7150/jca.52413

**Published:** 2021-01-01

**Authors:** Cailin Xue, Xudong Zhang, Peng Gao, Xiaohan Cui, Chunfu Zhu, Xihu Qin

**Affiliations:** 1 Department of Hepatobiliary Surgery, The Affiliated Changzhou No. 2 People's Hospital of Nanjing Medical University, Changzhou, Jiangsu 213003, P.R. China.; 2 Nanjing Medical University, Nanjing, Jiangsu, 211166, P.R. China.

**Keywords:** Hepatocellular carcinoma (HCC), long non-coding RNA (lncRNA), miR-30a-5p, SNAIL1, invasion, migration

## Abstract

**Background:** Hepatocellular carcinoma (HCC), a most common malignant tumor, has an unfavorable clinical outcome. Emerging evidence has demonstrated that long noncoding RNAs (lncRNAs) play an important role in the carcinogenesis and progression of HCC. However, the clinical significances and the biological roles of most lncRNAs in HCC remain poorly understood.

**Methods:** The expression levels of lncRNA loc339803 in HCC tissues and cell lines were determined by quantitative real-time polymerase chain reaction (qRT-PCR) assay. The cellular sublocalization of loc339803 was determined by fluorescence *in situ* hybridization and nuclear and cytoplasmic RNA isolation assay. Western blot, CCK-8, Edu, colony formation, migration and invasion assays were used to investigate the roles of loc339803 in HCC progression *in vitro*. A mouse model for lung metastasis was constructed to evaluate the role of loc339803 in HCC development *in vivo*. The correlations among loc339803, miR-30a-5p and SNAIL1 were validated by qRT-PCR and a dual- luciferase reporter assay.

**Results:** The expression of loc339803 was upregulated in HCC tissues and cell lines, and positively correlated with tumor size, advanced tumor stage, higher serum AFP level and poor prognosis of HCC patients. Loc339803 can promote the migration and invasion of HCC cells *in vivo* and *in vitro*. Further studies demonstrated that loc339803 functioned as a competing endogenous RNA (ceRNA) by directly binding to miR-30a-5p, thus up-regulating the expression of SNAIL1, a target gene of miR-30a-5p. Moreover, miR-30a-5p upregulation blocked the enhanced migration and invasion of HCC cells induced by loc339803 overexpression.

**Conclusions:** Loc339803 may be oncogenic in HCC and associated with poor clinical outcomes. LncRNA loc339803 might promote the invasion and migration of HCC cells through regulating miR-30a-5p/ SNAIL1 axis.

## Introduction

According to 2019 Global Cancer Statistics, hepatocellular carcinoma (HCC) is a most common malignant tumor with a fourth morbidity and a third mortality all over the world 1. Despite the advanced treatments, such as molecular targeted drugs, surgical resection, and radiofrequency ablation, the 5-year survival of HCC is still poor, usually due to the high rate of intrahepatic metastasis before diagnosis and the recurrence after surgical resection 2-6. Therefore, exploring the molecular mechanisms of HCC metastasis is urgently needed.

Long non-coding RNAs (lncRNAs), a type of RNA that is not longer than 200 nt, have no protein-coding ability 2. According to its transcription sites on the gene, lncRNAs can be divided into sense, antisense, intron, intergenic, and bidirectional lncRNAs 3. Numerous studies have showed that lncRNAs participate in the cell proliferation, invasion and apoptosis 4-6. The lncRNAs located in the nuclear and cytoplasm may exhibit different functions 11. As reported, lncRNAs located in the nucleus function as scaffolds to bring proteins to form ribonucleoprotein complexes and as guides to recruit chromatin-modifying complexes to target genes 6-8. The lncRNAs located in the cytoplasm function as competing endogenous RNAs (ceRNAs) to modulate the depression of miRNA targets through competitively binding miRNAs 9, 10. For instance, the TCONS_00006195 located in the nucleus can repress the progression of liver cancer by inhibiting the activity of ENO1 11. LncRNA MIR31HG located in cytoplasm can suppress the development of HCC by binding to miR-575 12. However, most lncRNAs remain poorly understood in tumorigenesis.

In present study, we selected a lncRNA from GEO datasets and explored its role in HCC. Our findings may provide a new therapeutic target for the treatment of HCC.

## Experimental Methods and Materials

### Clinical samples

A total of 80 pairs of liver cancer and the adjacent tissue samples were collected from Nanjing Drum Tower Hospital from 2018 to 2020. None of the patients had received chemotherapy and other treatments before surgery. Tissue specimens were placed in liquid nitrogen immediately after being collected and stored at -80 °C until use. The patients' pathological diagnoses were confirmed by two clinical pathologists. The basic clinical data and pathological grade of the patients are shown in Table 1. This study was approved by the Research Ethics Committee of Nanjing Drum Tower Hospital.

### Cell culture

The human HCC cell lines Hep3B, HepG2, SMMC-7721, LM3, 97H and LO2 were purchased from Cell Bank, Chinese Academy Sciences (Shanghai, China) and cultured in DMEM Dulbecco's Modified Eagle's Medium (DMEM), supplemented with 10% fetal bovine serum, 100U/ml penicillin, and 100 μg/ml streptomycin. All the cell lines were cultured in a humidified incubator with 5% CO_2_ at 37 °C.

### Transfection of cell lines

Small interference RNA (siRNA) and control for loc339803 were designed and synthesized by Hanbio gene (Guangzhou, China). Loc339803 full length (pCDH-loc339803) plasmid and controls (pCDH, Cat: CD510B-1) were synthesized by Sangon Biotech (Shanghai, China). MiR-30a-5p mimics, miR-30a-5p inhibitor and the control were designed and synthesized by GenePharma (Shanghai, China). HCC cells were transfected with the siRNA and plasmid following the lip3000 (Invitrogen, USA) manufacturer's instruction. The target sequences of loc339803 were listed as follows: si-loc339803#1: GGTGCGGTCTAGTGATCTA, si-loc339803#2: GCAAAGGTTTCTTATTTAA, and si-loc339803#3: GGTAGGAATCAAGGTTAGA. The target the sequences of snail1 were listed as follow: si-snail1: CCAGGCTCGAAAGGCCTTCA. The sequence of siloc339803#2 was subcloned into the lentiviral shRNA expression vector plko.1 (Addgene, USA). After the 48-hour transfection, the cells were harvested for the subsequent experiments.

### RNA extraction and RT-PCR detection

Total RNA from liver tissues and cells were extracted by using Trizol reagent (Invitrogen, Carlsbad, USA) following the instructions. Total RNA was reversely transcribed into cDNA using the First Strand cDNA Synthesis Kit (R232-01, Vazyme, Nanjing, China) and real-time PCR was carried out with the AceQ qPCR SYBR Green Master Mix (Q411-02, Vazyme, Nanjing, China). B-actin and U6 were used as the internal control. All the qPCR detections were performed using ABIQ6 (ABI, USA) and statistics were analyzed using the 2-ΔΔCt method. The primers used in this study are listed in Table 2.

### WB detection analysis

Total cell protein extraction was lysed with RIPA lysate containing 0.1% protease inhibitor and phosphorylase inhibitor, and quantified with BCA protein quantification kit. Subsequently, proteins were separated by SDS-PAGE and transferred to PVDF membranes. The PVDF membranes were blocked with 5% milk at room temperature for 2 hours, and then incubated with corresponding primary antibodies at 4 °C overnight. The following antibodies were used for western blot: E-cadherin (Cat: #3195T, CST), N-cadherin (Cat: #13116T, CST), Vimentin (Cat: #5741T, CST), MMP-9 (Cat: #13667, CST), MMP2 (Cat: #40994, CST), GAPDH (Cat: #5174, CST), SNAIL1 (Cat: #3879, CST). After incubation with a secondary antibody at room temperature for 2 hours, the membranes were imaged with ECL (Tanon, Shanghai, China) luminescent solution.

### CCK8 and Edu proliferation assay

For proliferation experiment, 4,000 cells were planted in 96-well plates and 3 wells were used for technical replicates. 100 ul DMEM of solution containing 10% CCK8 (Dojindo Laboratories, Kumamoto, Japan) was added into each well. After being cultured for 2 hours in a humidified incubator, the absorbance was measured at 450 nm wavelength. A Cell-Light Edu DNA cell proliferation kit (Ribobio, Guangzhou, China) was used to evaluate the cell proliferation ability.

### Clone formation experiment

For the colony formation assay, 600 cells were seeded in 6-well-plates and cultured with DMEM containing 10% FBS for 2 weeks. The colonies were fixed with 4% paraformaldehyde for 20 minutes, and then stained with crystal violet for 5 minutes at room temperature.

### Invasion and migration assays

The cell lines were harvested after transfection with siRNA or plasmids for 48 hours. 5×10^4^ cells were resuspended in DMEM medium (200 μL), and then seeded in the top chamber of transwell plates (8 μm size, Corning, NY, USA). For invasion, the chamber was coated with 30 ug Matrigel, while for the migration assay; the insert was not coated with Matrigel. The lower chamber was supplemented with 1ml DMEM containing 10% FBS. The plates were cultured in a humidified incubator with 5% CO_2_ at 37 °C for 12 hours. The cells that had migrated and invaded were fixed with 4% paraformaldehyde, stained with crystal violet, and then counted. The pictures were taken by a 200× microscope (Olympus, Japan).

### *In vitro* experiments

For metastasis experiment *in vivo*, 5×10^6^ Hep3B cells which had been infected with shRNA-loc339803 or empty vector were resuspended in 300 μL PBS, and injected into 4-week-old BALC nude mice through the tail vein (5 mice per group). The experiment was terminated 8 weeks after the tail vein injection. The lungs were dissected, fixed with 4% paraformaldehyde, and prepared for histological examination.

### FISH experiment and nuclear-plasma separation

The loc339803, U6 and 18S fish probes were designed and synthesized by Guangzhou Ribobio Co, Ltd. (Guangzhou, China). The fish excrement was carried out by using the fish kit according to the manufacturer's instructions. The images were acquired through confocal microscope (Leica).

### Nuclear and cytoplasmic RNA isolation assay

The total, cytoplasm and nuclear RNA were isolated using a Cytoplasmic and Nuclear RNA Purification Kit according to the manufacturer's instruction (Norgen Biotek, Canada). U6 acted as nuclear internal control, and 18s acted as cytoplasmic internal control. Data normalization was performed against total RNA: % of Input = 100 × (2^[Ct total RNA-Ct RNA fraction]^.

### Luciferase reporter gene experiment

3′-UTR of SNAIL or loc399803 was inserted into pGL3 luciferase reporter vector (Promega, Madison, WI, USA). 293 T cells were transfected with 0.5 ug reporter construct and 50 nmol siRNA (or miRNA mimic) per well using Lipofectamine 3000 (Invitrogen, Cat#L3000-015). After 12 hours of transfection, the transfection medium was replaced by complete culture medium. After 48 hours of culture, the cells were lysed with passive lysis buffer (Promega, Cat# E1910), and the reporter gene expression was assessed using a Dual Luciferase reporter assay system (Promega, Cat# E1910). All transfection assays were carried out in triplicate.

## Experimental Results

### Loc33983 was up-regulated in HCC and positively correlated with the prognosis of the patient

**Figure 1:**
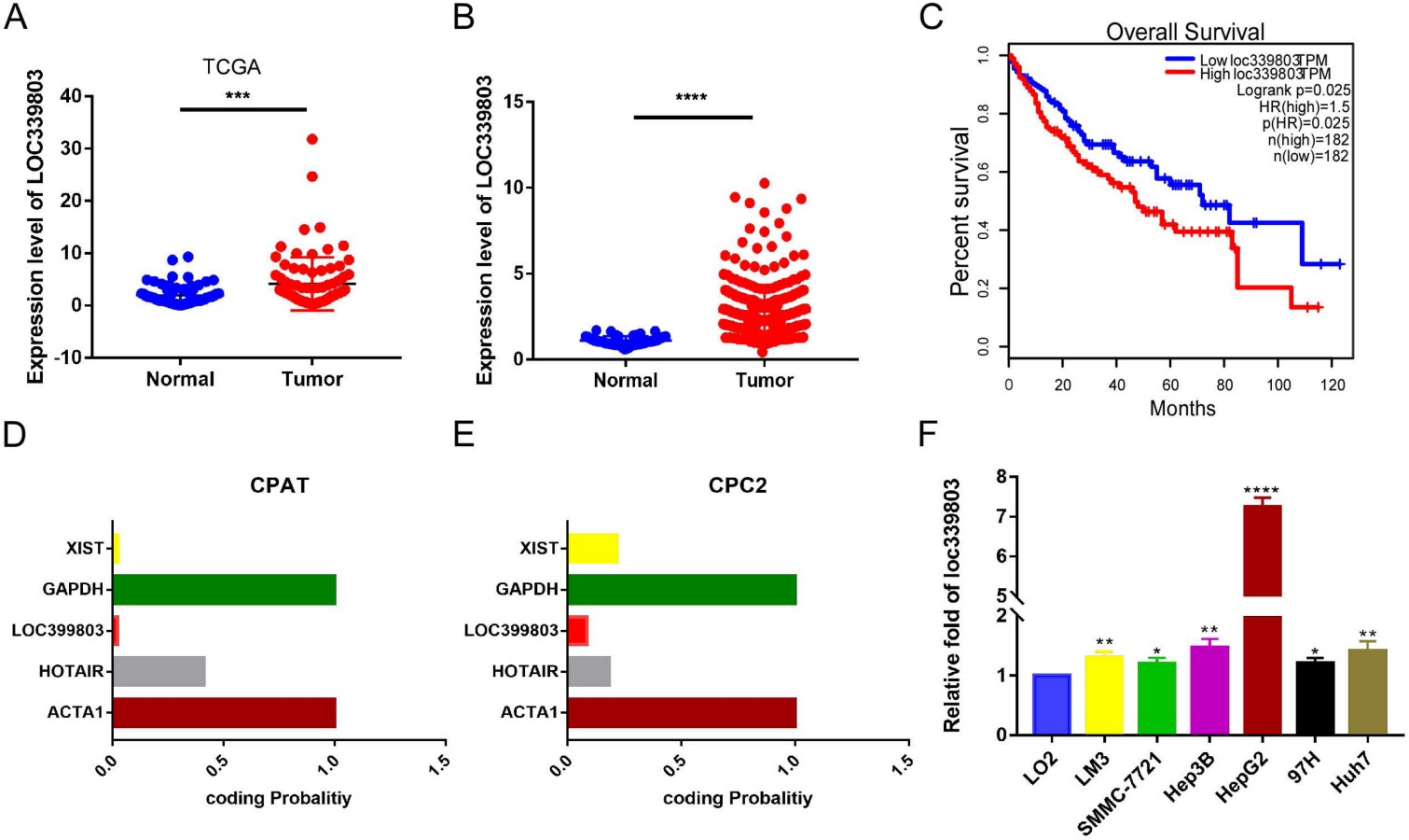
** Loc339803 is upregulated in HCC and have a significant correlation with the outcome of the patients. A.** TCGA dataset in starbase 3.0 indicated the loc339803 is high expressed in HCC compared to normal tissue (*P* < 0.0001, N=86). **B.** The expression of loc339803 in 85 pairs of HCC and matched precancerous tissues was measured by qRT-PCR (*P* = 0.0002, N=85). **C.** Survival analysis of TCGA data from starbase 3.0 relevel that the higher loc339803 expression indicated a poorer survival outcome. The median expression level of loc339803 was used as the cut-off; *P* = 0.0025. **D, E.** The coding ability of loc339803 was evaluated in CPAT and CPC2. **F.** Loc339803 was widely overexpression in HCC cell lines.

To discover abnormally expressed lncRNA in liver cancer tissues, we searched the GEO database and identified a large number of differentially expressed lncRNAs in GSE59747 dataset 13. Among them, the lncRNA loc339803 which was upregulated 2.33-flod by the TGF-β attracted our attention (Table S1). Then we investigated loc339803 by analyzing the TCGA data in Starbase 3.0 14. The lncRNA loc339803 was highly expressed in HCC and had a positive correlation with the survival time of the patients (Figure 1A,C). We further examined 86 pairs of tumor and matched adjacent peritumoral tissues. The lncRNA loc339803 was upregulated in tumor tissues (Figure 1B). The loc339803 was also upregulated in HCC cell lines Hep3b, SMMC-7721, Lm3,97h, and HepG2, compared with normal liver cell line LO2 (Figure 1F). Through analyzing the coding ability of loc339803 in CAPT and CPC2 databases, we found that loc339803 had a poor protein coding ability (Figure 1D,E).

Furthermore, to assess the clinical significance of loc339803 in HCC, we analyzed the correlation between the loc399803 expression level and the clinal outcome of HCC patients. The results showed that the patients with a higher expression of lco399803 had a higher degree of malignancy and a higher level of serum AFP (Table 1).

### Loc339803 promoted the invasion and migration of HCC *in vitro*

**Figure 2:**
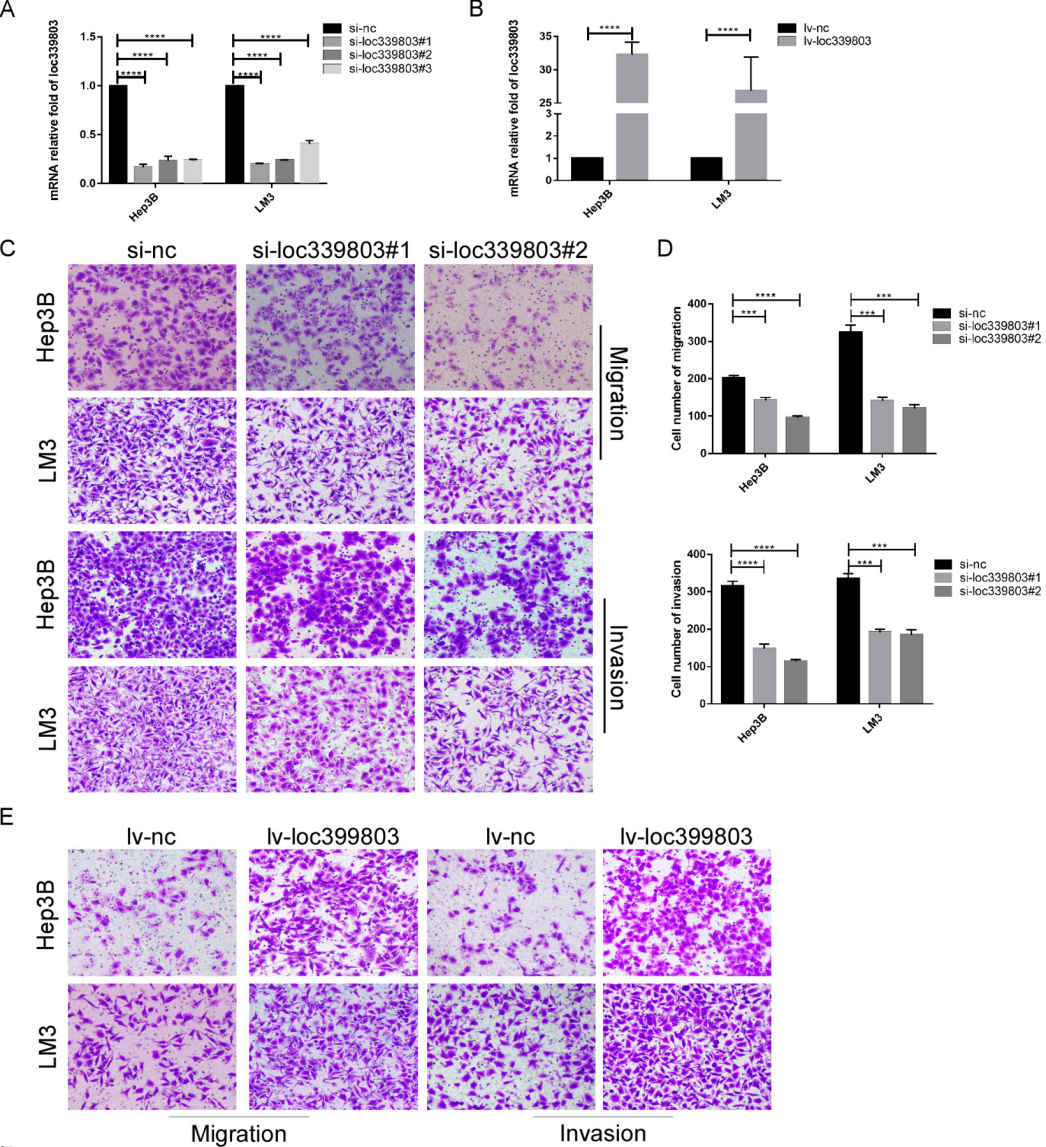

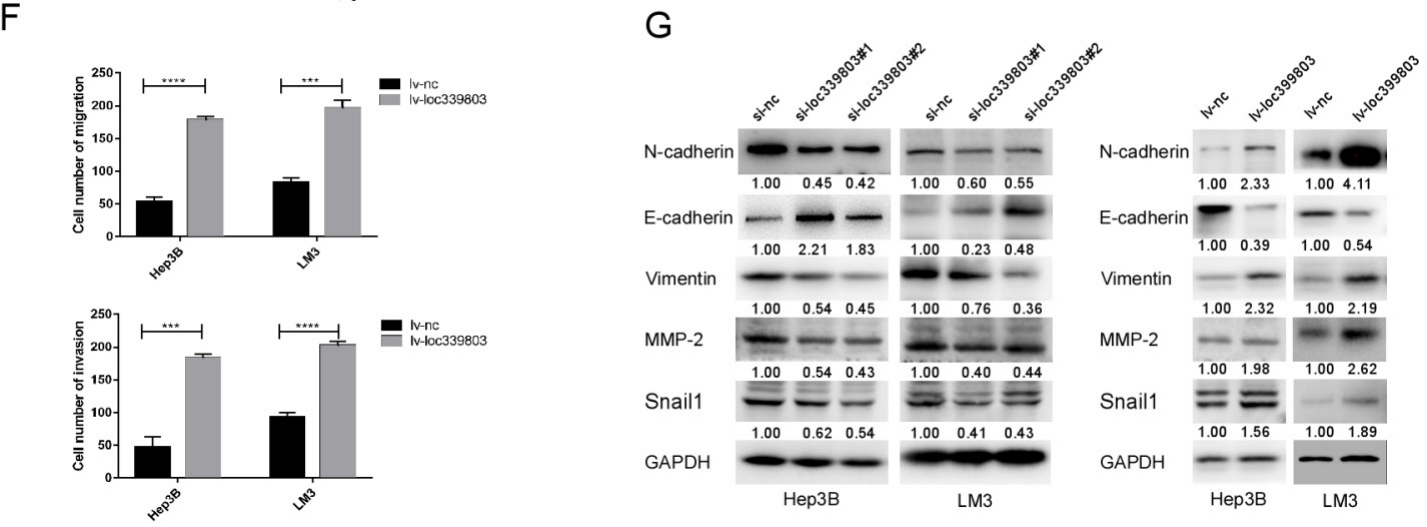
**Loc339803 promote migration and invasion of HCC cell both *in vitro*. A.** The interference effect of siRNA against loc339803. **B.** The loc339803 expression can be overexpressed by Lv-loc339803. **C,D.** Transwell analysis indicated knowdown loc339803 can inhibit the invasion and migration of HCC cells in hep3B and LM3. **E,F.** The migration and invasion ability of HCC cell can be enhanced by overexpressed loc339803. **G.** Western blot show loc339803 can promoted the expression of N-cadherin, Vimentin, MMP-2 and snail 1.

To further investigate the function of loc339803 in HCC, liver cancer cells Hep3B and LM3 were selected to perform loss- and gain-of function experiments. First, three siRNA oligos were transfected into Hep3B and LM3. The knockdown efficiency was showed in Figure 2A. The overexpression efficiency was showed in Figure 2B. The transwell assay showed the ability of migration and invasion of Hep3b and LM3 was reduced after loc339803 knockdown (Figure 2C,D) and enhanced by loc339803 overexpression (Figure 2E,F). The cck8, EDU and cloning formation assay showed the loc339803 had no effect on the prefiltration of Hep3b and LM3 (Figure S1). The western blot results showed that loc339803 increased the expression N-cadherin, Vimentin, MMP-2, SNAIL1 and decreased the E-cadherin expression (Figure 2G).

### Loc339803 promoted the invasion and migration of HCC *in vivo*

**Figure 3:**
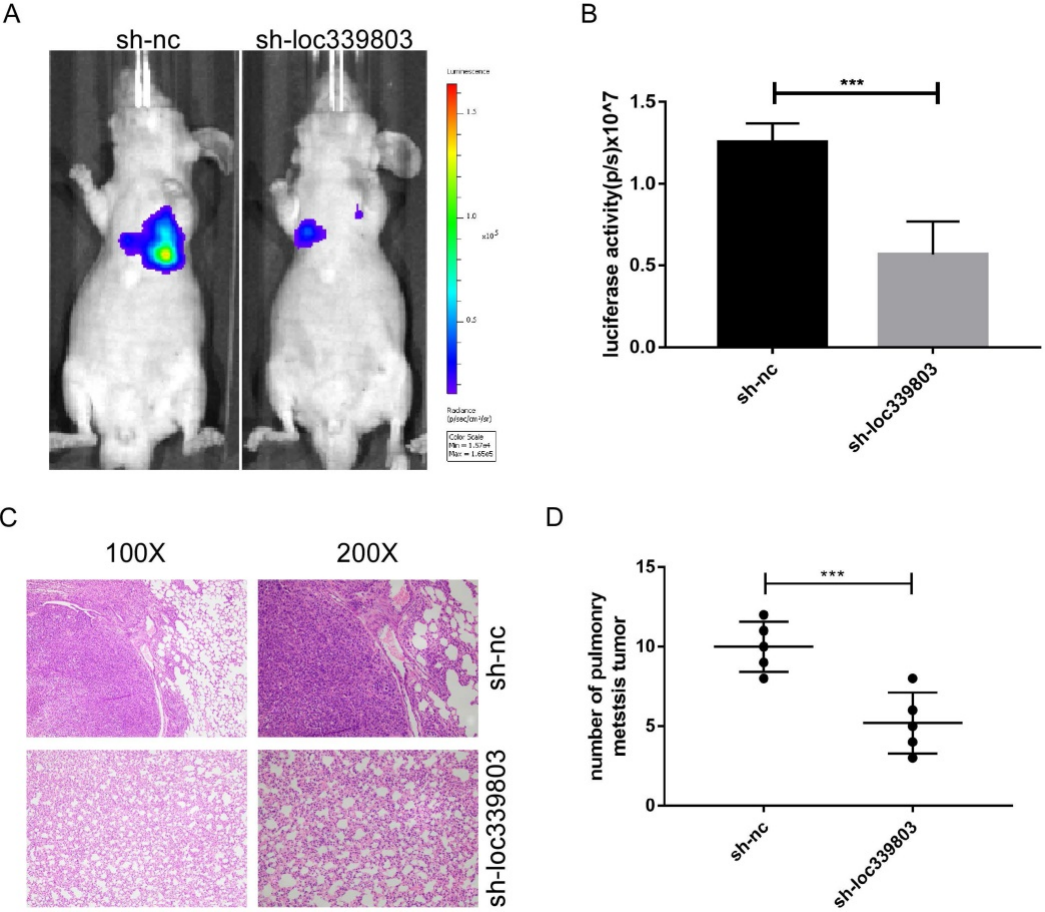
** Loc339803 can promote the invasion and migration *in vivo*. A,B.** Sh-loc339803 or sh-nc hep3B cell were injected into nude mice through tail vein (N=5), and the luciferase signal intensities of the group were examined at 8 weeks (*p*=0.002). **C,D.** The representative HE and the number of lung metastatic tumors show that loc339803 can promote the pulmonary metastatic of HCC (*p*=0.003).

To investigate the role of loc339803 in tumor metastasis *in vivo*, the lung metastases model was established. The Hep3B cells stably transfected with sh-loc339803 or sh-nc were injected into nude mice through tail vein (5 mice per group). Eight weeks later, the lung metastasis foci were detected with a live imaging system. The bioluminescence showed loc339803 knockdown could significantly inhibit the lung metastases (Figure 3A,B). Histological examination of the lung showed the mice injected with sh-loc339803 cells have fewer pulmonary metastatic nodules compared with the sh-nc group (Figure 3C,D).

### Loc339803 functioned as ceRNA and sponged miR-30a-5p in HCC cell lines

**Figure 4:**
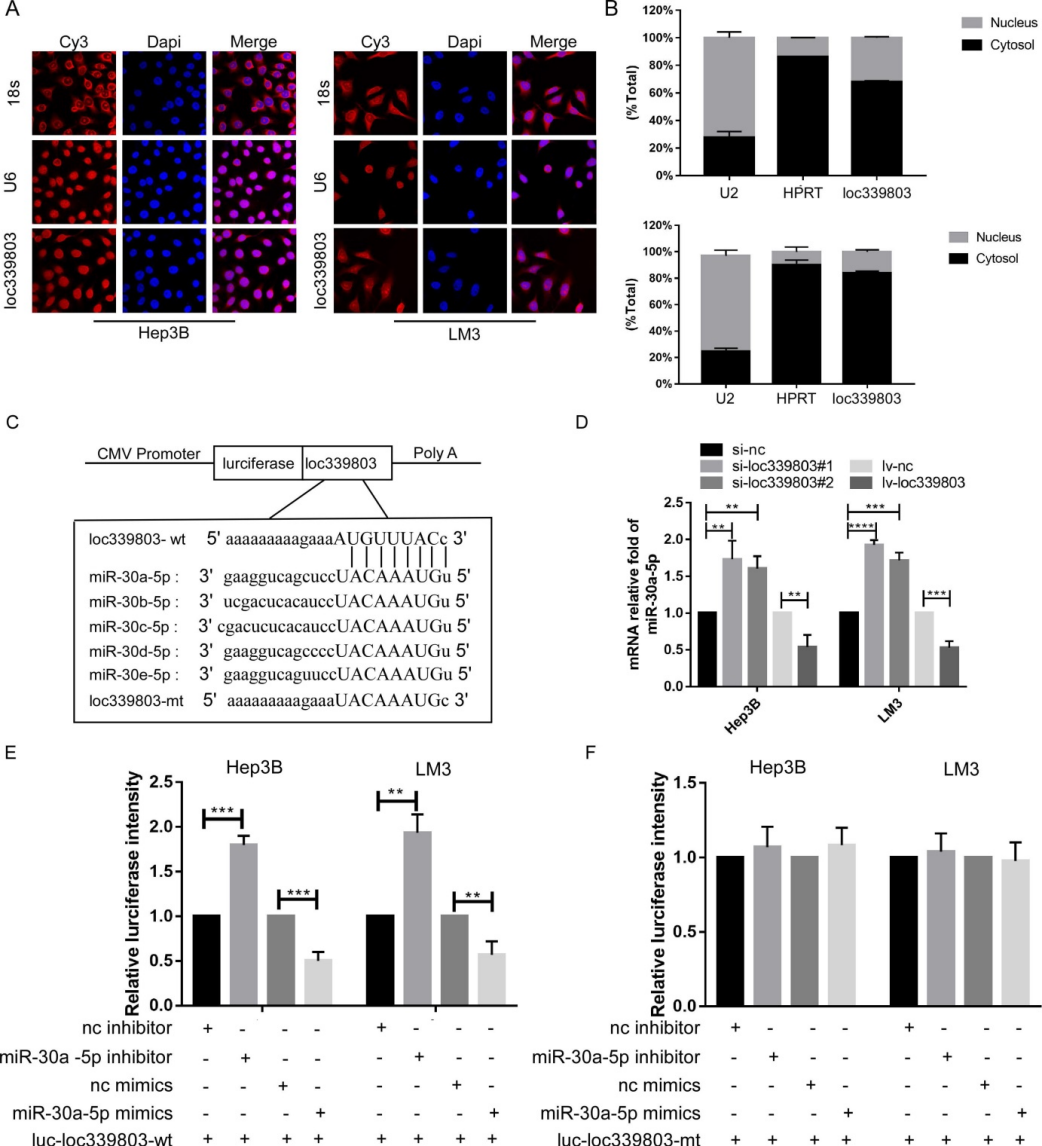
** loc339803 functions as a molecular sponge of miR-30a-5p in HCC cells. A.** Fish-experiment exhibited that the loc339803 is mainly located in cytoplasm. **B.** Nuclear & cytoplasmic RNA isolation assay also show that exhibited that the loc339803 is mainly located in cytoplasm. **C.** Bioinformatics predicts that miR-30a/b/c/d/e-5p can bind to the sequence of loc339803. **D.** The overexpression of loc339803 can reduce the expression of miR30a-5p, while knockdown loc399803 can promote the expression of miR-30a-5p. **E.** Luciferase reporter gene showed that miR-30a-5p reduced luciferase activity of the wild type loc339803. **F.** Luciferase reporter gene showed that miR-30a-5p cannot reduce luciferase activity of the mutation type loc339803.

Evidence has indicated that lncRNA can bind to protein in nuclear and sponge on miRNA to regulate the expression level of target gene in cytoplasm. To investigate the mechanism through which loc339803 regulated migration and invasion, we analyzed the location of loc339803. The lncRNA location database (lncLocator), the Fish-experiment and Nuclear and cytoplasmic RNA isolation assay exhibited that the loc339803 was mainly located in cytoplasm (Figure 4A,B), indicating that loc339803 might function as ceRNA. Then, we investigated miRNA that might bind to loc339803 in starbase v3.0, and miRNAs 30a/b/c/d/e were identified to bind to loc339803 (Figure 4C). Thus, we examined the expression of miR-30a/b/c/d/e after loc339803 low- and over-expression (Figure S2). We found that the expression of miR-30a-5p was significantly decreased by loc339803 overexpression compared to miR-30b/c/d/e, and increased by loc339803 knockdown (Figure 4D). Furthermore, the luciferase reporter assay revealed that overexpression of miR-30a-5p reduced the luciferase activity of wild-type loc339803 vector in Hep3B and LM3 (Figure 4E). The luciferase activity of wild-type loc339803 could be upregulated by inhibiting the miR-30a-5p expression (Figure 4F).

### SNAIL1 was a target of miR-30a-5p in HCC cell lines

**Figure 5:**
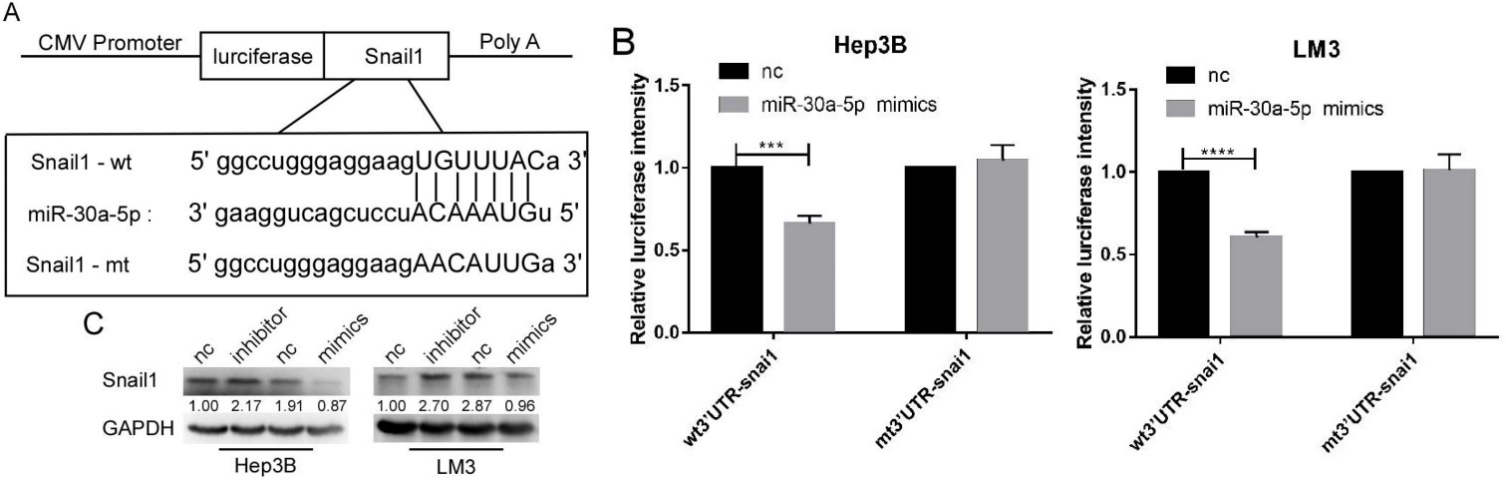
** Snail1 is a target gene of miR-30a-5p. A.** The bioinformatics prediction that miR-30a-5p can target the 3UTR region of snail1. **B.** Luciferase reporter gene showed that miR-30a-5p could significantly reduce the luciferase activity of wild type snail1. **C.** Western blot shows the expression of snail1 can be regulated by miR-30a-5p.

By the analysis based on starbase v3.0, we found that SNAIL1 was a target of miR-30a-5p (Figure 5A). The luciferase activation was reduced by miR-30a-5p mimics (Figure 5B). Further, we found the expression level of SNAIL1 was downregulated when miR-30a-5p was highly expressed, and upregulated when miR-30a-5p was lowly expressed (Figure 5C).

### MiR-30a-5p restoration attenuated the effect of loc339803 overexpression on HCC cells

**Figure 6:**
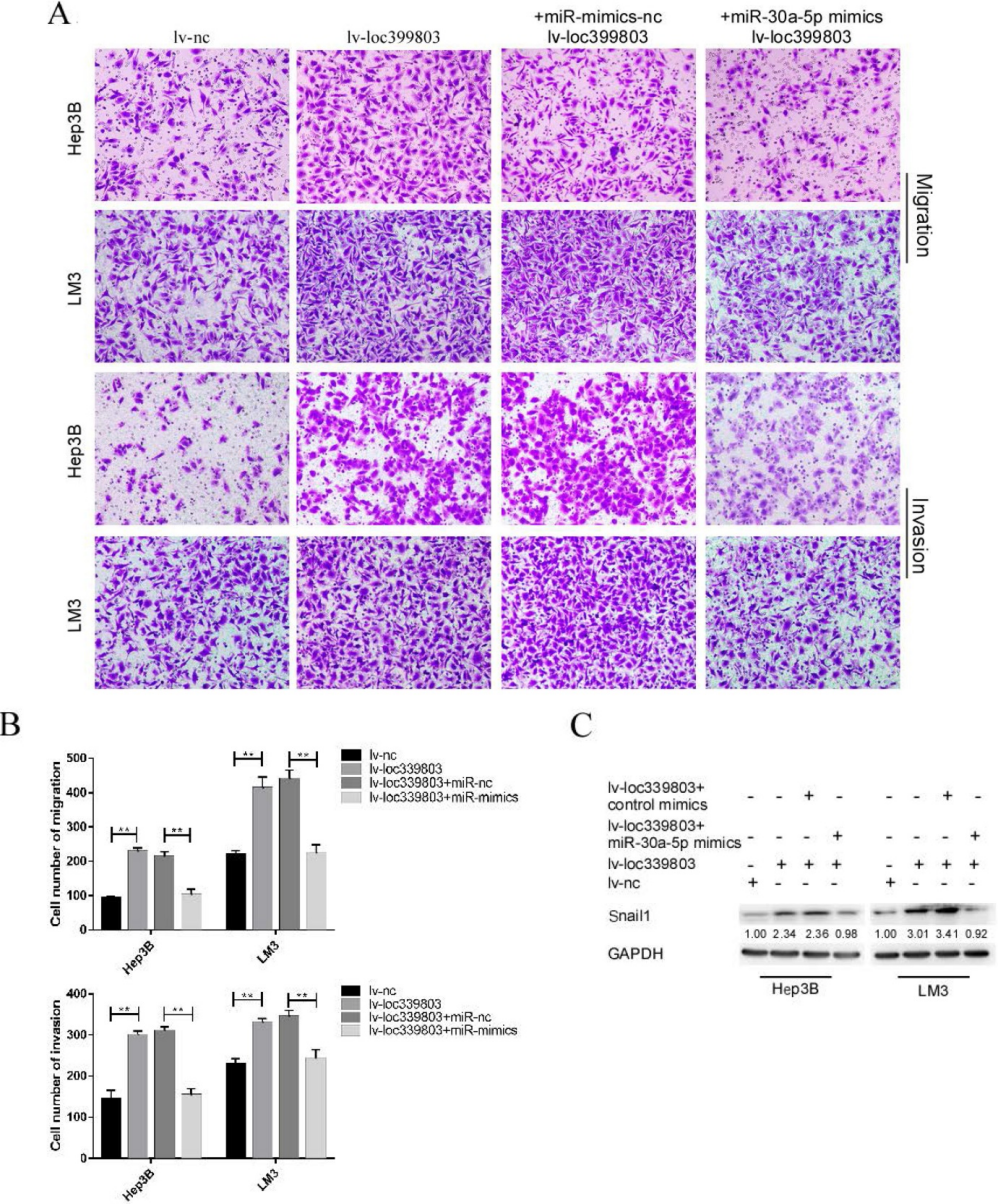
** miR-30a-5p can block loc339803-induced tumor invasion and migration. A,B.** Loc339803 can promote the migration and invasion of HCC cell lines Hep3B and LM3. Additionally, overexpression of miR-30a-5p can block the enhanced invasion and migration ability of cells induced by loc339803. **C.** MiR-30a-5p was able to restore the over-expression of snail1 which enhanced by loc339803.

After transfection of miR-30a-5p mimics with stably overexpressed loc399803, we found that the loc39983-enhanced invasion of HCC was blocked by miR-30a-5p (Figure 6A). The loc399803-induced upregulation of SNAIL1 was blocked by miR-30a-5p mimics. In HCC cell lines with loc339803 overexpression, the enhanced migrative and invasive abilities were partly abolished by miR-30a-5p overexpression (Figure 6B).

### SNAIL1 knockdown blocked the loc339803-induced enhancement of migration and invasion

**Figure 7:**
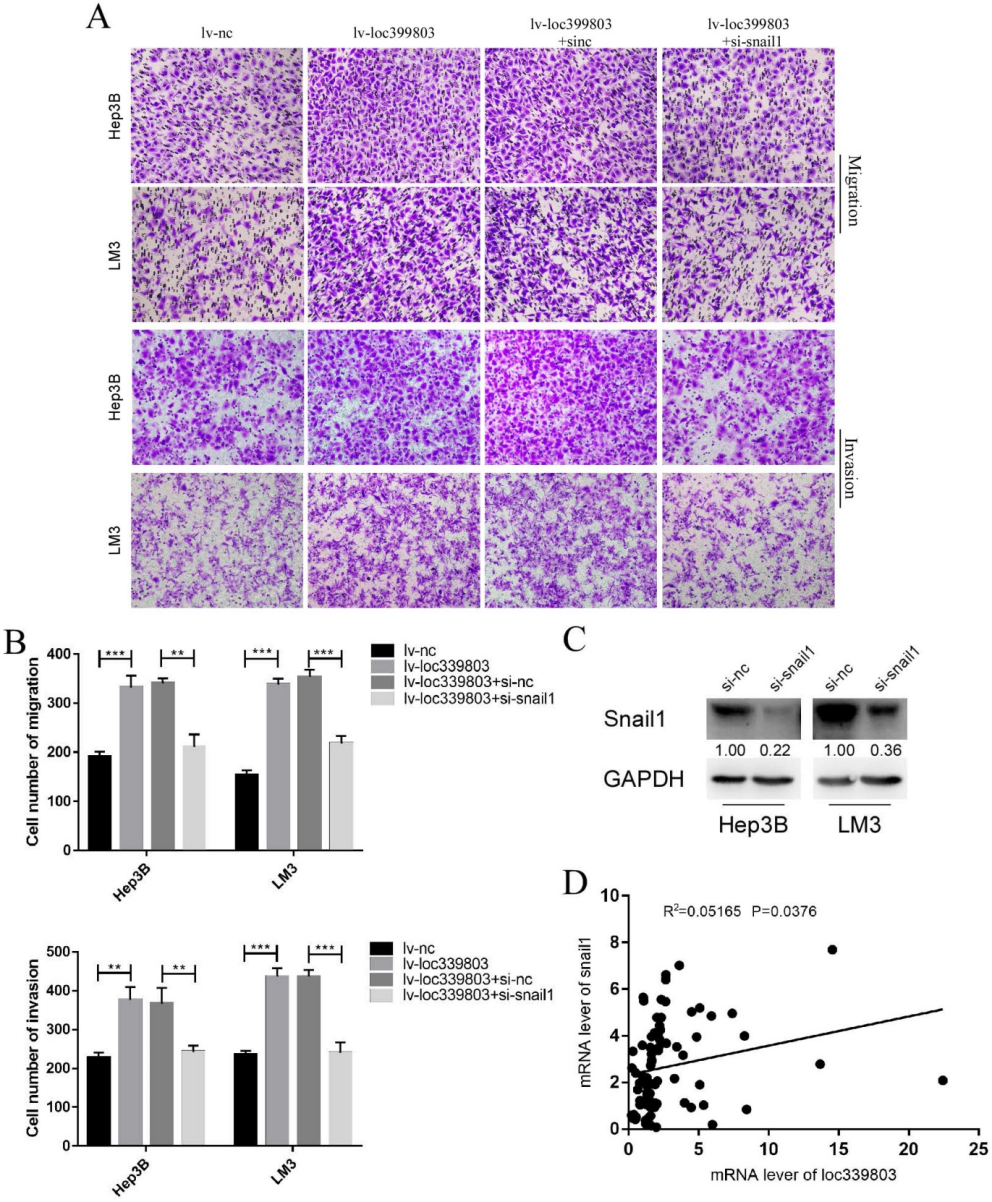
** Knockdown the expression of snail1 can block loc339803-induced tumor invasion and migration. A,B.** Loc339803 can promote the migration and invasion of HCC cell lines Hep3B and LM3. Further, downregulation the expression of snail1 can block the enhanced invasion and migration ability of cells induced by loc339803. **C.** The siRNA targeting snail1 can significantly reduce the expression of snail1 in Hep3B and LM3. **D.** The mRNA of loc339803 significantly correlates with the mRNA of snail1 in 84 cases tumor and match tissues.

Through analyzing the mRNA of c and loc339803 in the tumor and adjacent tissues, we found that there was a high correlation between SNAIL1 and loc339803 (Figure 7D). In the loc339803 overexpression cell lines, when SNAIL1 was knocked down (Figure 7C), the ability of migration and invasion induced by the loc339803 overexpression became weaker (Figure 7A,B).

## Discussion

Emerging studies have shown that lncRNAs take part in the development of cancer 15, 16. In this study, we found that loc339803, a newly discovered lncRNA, was highly expressed in HCC tissues and positively associated with the grade of the HCC and the level of AFP in serum. Besides, HCC patients with a higher level loc339803 showed worse outcomes and shorter survival time. These results showed that the loc339803 might play an important role in the progress of HCC and therefore, was worth in-depth investigation.

More and more study indicated that the epithelial-mesenchymal transition (EMT) plays an important role in the migration and invasion of cancer cells. EMT refers to the transformation of epithelial cells into mesenchymal cells, even malignant phenotype, which contributes to tumor metastasis 17, 18. N-cadherin, Vimentin, and E-cadherin are the biomarkers of EMT. SNAIL1 is a transcription factor which facilitates EMT. So far, lots of lncRNAs have been confirmed to be involved in EMT. For example, lncTCF7 and SNHG3 can promote the progress of cancer through enhancing EMT in HCC 19, 20. In this study, we discovered that the loc339803 enhanced the migration and invasion of HCC cell lines *in vivo* and *in vitro*, and loc339803 overexpression upregulated N-cadherin, Vimentin, SNAIL1 and downregulated E-cadherin. Besides, inhibiting the expression of SNAIL1 can block the migration and invasion of HCC cells induced by loc339803 upregulation. These results indicated that loc339803 might promote EMT of HCC cell lines by upregulation of SNAIL1.

The function of lncRNA is determined by its location in the cells 21, 22. LncRNA located in the cytoplasm can function as ceRNA by competitively binding to miRNA, thereby affecting the expression level of miRNA-targeted genes 23, 24. In this study, the location of loc339803 was predicted to be in the cytoplasm according to the analysis of the lncLocator database 25. Furthermore, the follow experiments verified the results. Thus, we believed that loc339803 might function as ceRNA to promote the migration and invasion of HCC cell lines. Bioinformatics assays and the luciferase reporter gene tests then confirmed miR-30a-5p as a target miRNA of loc339803. MiR-30a-5p can act as a suppressor gene to inhibit the migration and invasion in many cancers 26-28. In this study, we found the migration and invasion of HCC cells, which had been enhanced by loc339803 overexpression, was blocked by miR-30a-5p mimics. And the expression of miR-30a-5p could be suppressed by loc339803 overexpression. Therefore, miR-30a-5p could be a target gene of loc339803.

MiRNA can reduce target-gene expression by binding with 3-UTR of mRNA. The lncRNA- miRNA-mRNA axis has been reported to take part in cancer progression. The bioinformatic assays indicated SNAIL1 may be a target gene of miR-30a-5p. The luciferase reporter gene tests and the western blot assay also verified the results. In addition, SNAIL1 has been confirmed as a target of miR-30a-5p in lung cancer and stomach cancer, and a regulator in EMT 29, 30. In the following experiments, we found SNAIL1 was regulated by miR-30a-5p in HCC. The luciferase reporter gene tests also showed that miR-30a-5p could bind to SNAIL1. Taken together, loc339803 could positively regulate the expression of SNAIL1 via targeting miR-30a-5p in HCC cells.

## Conclusion

In summary, loc339803 is highly expressed in HCC and has a positive correlation with the poor prognosis of patients. Loc339803 promotes the invasion and migration of HCC cells by sponging on miR-30a-5p. Our study provides new insights into the molecular mechanism of loc339803 in promoting HCC metastasis. Loc339803 may be a potential target for the treatment of liver cancer.

## Supplementary Material

Supplementary figures and tables.

## Figures and Tables

**Table 1:** Correlation of HCC clinicopathologic features and loc339803 expression in tumor tissues (N=85)

Characteristics	Loc339803 level		*P* value
	High expression	Low expression	
Case	43	42	
**Age (years)**			0.258
<60	25	30	
≥60	18	12	
**Gender**			
Man	31	29	0.815
Female	12	13	
**Cirrhosis**			
Yes	22	24	0.665
No	21	18	
**Size**			
<5	20	18	0.828
≥5	23	24	
**TNM**			
I-II	20	30	0.028*
III-IV	23	12	
**AFP (ng/ml)**			
<200	18	29	0.016*
≥200	25	13	

According to the mean value of loc339803 in the tumor tissues of 85 HCC patients, we divide all the patients into two group: loc339803 high expression group (n = 43) and loc339803 low expression group (n = 42). *P < 0.05.

**Table 2:** The primer used in this study

Gene name	Primer sequences (5' to 3')	
hsa-miR-30a-5p	sense	ggggTGTAAACATCCTCGACT
	anti-sense	CAGTGCGTGTCGTGGAGT
hsa-miR-30b-5p	sense	ggggTGTAAACATCCTACACT
	anti-sense	CAGTGCGTGTCGTGGAGT
hsa-miR-30c-5p	sense	ggggTGTAAACATCCTACACTC
	anti-sense	CAGTGCGTGTCGTGGAGT
hsa-miR-30d-5p	sense	gggTGTAAACATCCCCGACT
	anti-sense	CAGTGCGTGTCGTGGAGT
hsa-miR-30e-5p	sense	ggggTGTAAACATCCTTGACT
	anti-sense	CAGTGCGTGTCGTGGAGT
LOC339803	sense	AGTGATTATGACCCGTGA
	anti-sense	TGAGTTGCTCCATCTTTC
RT-primer for miRNA		GTCGTATCCAGTGCGTGTCGTGGAGTCGGCAATTGCACTGGATACGAC
GAPDH	sense	CAGGAGGCATTGCTGATGAT
	anti-sense	GAAGGCTGGGGCTCATTT
U2	sense	ATACGTCCTCTATCCGAGGACA
	anti-sense	TGGAGGTACTGCAATACCAGGT
HPRT	sense	AGGCCATCACATTGTAGCCC
	anti-sense	CAACACTTCGTGGGGTCCTT
