# Supplementary figures and images for: LncRNA loc339803 acts as CeRNA of miR-30a-5p to promote the migration and invasion of hepatocellular carcinoma cells
Source: J Cancer. 2021 Jan 1;12(4):1061–72. doi: 10.7150/jca.52413 (PMC7797647; doi:10.7150/jca.52413)

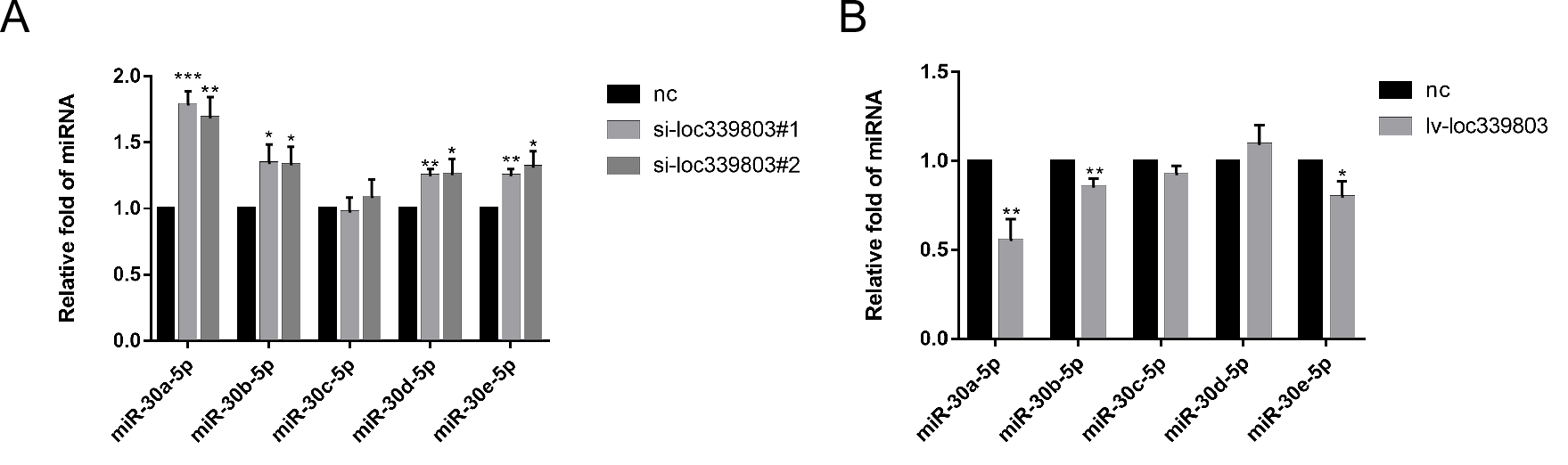

Supplement: Supplementary file 1 — Supplementary figures and tables. [file jcav12p1061s1.zip › Figure S2.tif]

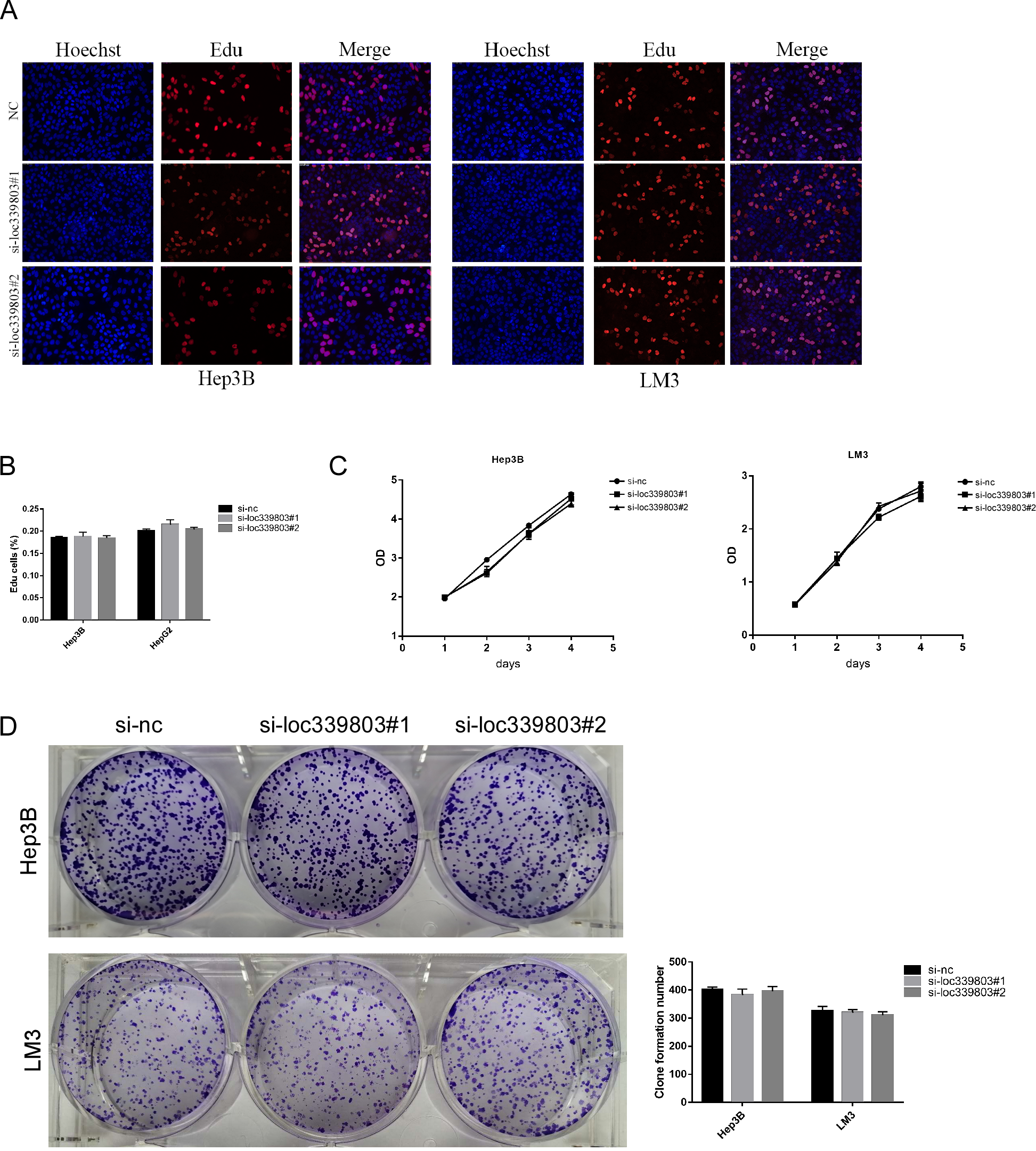

Supplement: Supplementary file 1 — Supplementary figures and tables. [file jcav12p1061s1.zip › Figure S1.tif]
