# Supplementary material for: LncRNA loc339803 acts as CeRNA of miR-30a-5p to promote the migration and invasion of hepatocellular carcinoma cells
Source: J Cancer. 2021 Jan 1;12(4):1061–72. doi: 10.7150/jca.52413 (PMC7797647; doi:10.7150/jca.52413)
Supplement: Supplementary file 1 — Supplementary figures and tables. [file jcav12p1061s1.zip › Table s1.doc]

| **seqname** | **p-value** | **Fold change** | **regulation** |
| --- | --- | --- | --- |
| ENST00000444114 | 0.000189599 | 20.281694 | up |
| AL359062 | 0.000440182 | 17.087671 | up |
| BC002831 | 0.000681707 | 13.281824 | up |
| ENST00000510327 | 0.000419314 | 12.5515375 | up |
| uc003yqp.1 | 0.009352703 | 9.988064 | up |
| ENST00000423408 | 9.27853E-05 | 7.6446257 | up |
| ENST00000458282 | 0.003236673 | 6.8852134 | up |
| CR612147 | 0.00035125 | 5.690169 | up |
| AK021425 | 0.009098443 | 5.019492 | up |
| uc001vqh.2 | 0.000751036 | 4.736723 | up |
| M31164 | 0.005721103 | 4.6642694 | up |
| AK022299 | 0.006683667 | 4.2585044 | up |
| ENST00000516126 | 0.000641661 | 4.1110888 | up |
| BC002465 | 0.04266339 | 4.0987787 | up |
| ENST00000411949 | 0.001431148 | 3.9110913 | up |
| uc010bol.1 | 0.04797761 | 3.9081898 | up |
| uc001gla.1 | 0.000447058 | 3.292722 | up |
| uc001eky.3 | 0.003489965 | 3.2678733 | up |
| ENST00000430494 | 0.001028988 | 3.2129085 | up |
| uc010pbe.1 | 0.003483687 | 3.111105 | up |
| AK092850 | 0.000858404 | 3.0965269 | up |
| ENST00000438453 | 0.012970074 | 3.0890198 | up |
| BC041955 | 0.004214332 | 3.0492406 | up |
| ENST00000406468 | 0.035726044 | 3.0479548 | up |
| NR_026813 | 0.033064377 | 3.0409381 | up |
| ENST00000508851 | 0.003097615 | 3.0397081 | up |
| ENST00000493038 | 0.04425564 | 3.0262718 | up |
| ENST00000429139 | 0.018040236 | 3.0164628 | up |
| ENST00000429139 | 0.018040236 | 3.0164628 | up |
| uc004exm.2 | 0.000174698 | 2.9978557 | up |
| AK024197 | 0.02917039 | 2.9924982 | up |
| NR_033354 | 0.015201448 | 2.989216 | up |
| AK095302 | 0.042796798 | 2.985813 | up |
| U79293 | 0.011519754 | 2.985041 | up |
| ENST00000501836 | 0.003291078 | 2.9657502 | up |
| ENST00000456887 | 0.002112135 | 2.9389625 | up |
| NR_027145 | 0.016639208 | 2.9025657 | up |
| ENST00000512617 | 0.005109306 | 2.8983305 | up |
| uc001xfp.2 | 0.007012097 | 2.8884802 | up |
| AK125090 | 0.029146122 | 2.8748841 | up |
| ENST00000511779 | 0.006825061 | 2.860412 | up |
| uc002zkr.3 | 0.04626502 | 2.8306754 | up |
| NR_003661 | 0.023462621 | 2.819027 | up |
| ENST00000421730 | 0.035720292 | 2.8173327 | up |
| uc001ich.2 | 0.018976301 | 2.7691886 | up |
| AK027383 | 0.030779785 | 2.7644799 | up |
| uc003adh.2 | 0.013547535 | 2.7644188 | up |
| AF052107 | 0.024511736 | 2.7380674 | up |
| AK021877 | 0.03839913 | 2.735031 | up |
| ENST00000483283 | 0.039488204 | 2.7232761 | up |
| BC008085 | 0.044175364 | 2.7182057 | up |
| AF074982 | 0.007667336 | 2.7105064 | up |
| CR612226 | 0.020072805 | 2.7046108 | up |
| AK123959 | 0.005169846 | 2.7011375 | up |
| uc002tel.2 | 0.003362201 | 2.6919112 | up |
| AF176921 | 0.003238504 | 2.6859052 | up |
| NR_002569 | 0.001320258 | 2.5668776 | up |
| ENST00000510453 | 0.00409379 | 2.5620775 | up |
| uc001rjo.1 | 0.003035022 | 2.5613189 | up |
| AK127902 | 0.04175838 | 2.5194776 | up |
| AK127902 | 0.04175838 | 2.5194776 | up |
| DB314527 | 0.00247062 | 2.515921 | up |
| ENST00000411291 | 0.007050654 | 2.5061898 | up |
| HIT000067091 | 0.008671412 | 2.49469 | up |
| ENST00000398170 | 0.033089142 | 2.4922373 | up |
| ENST00000428211 | 0.019317804 | 2.4784632 | up |
| AK129540 | 0.001429717 | 2.4761424 | up |
| uc003tvu.2 | 0.046155244 | 2.4737864 | up |
| uc004bos.2 | 0.006727519 | 2.4501636 | up |
| NR_024368 | 0.021930689 | 2.4418695 | up |
| ENST00000428667 | 0.026823003 | 2.441584 | up |
| ENST00000476892 | 2.35041E-05 | 2.433771 | up |
| ENST00000415869 | 0.020870987 | 2.42452 | up |
| NR_027467 | 0.000501248 | 2.4200866 | up |
| AK127847 | 0.013070744 | 2.4198797 | up |
| BX649150 | 0.01766644 | 2.4181437 | up |
| NR_033313 | 0.017947337 | 2.401694 | up |
| uc002hng.1 | 0.017549125 | 2.386749 | up |
| ENST00000455105 | 0.019831344 | 2.372253 | up |
| G36947 | 0.026545845 | 2.3668644 | up |
| AK023447 | 0.044898517 | 2.359542 | up |
| ENST00000505389 | 0.00814423 | 2.3556383 | up |
| BC013423 | 0.00693279 | 2.3441849 | up |
| ENST00000417691 | **0.014678516** | **2.33432** | **up** |
| AK095165 | 0.019669969 | 2.3329124 | up |

**Table s1**

The representative elevated lncRNA in GSE59747
